# Cephalometric Evaluation of Children with Short Stature of Genetic Etiology: A Review

**DOI:** 10.3390/children11070792

**Published:** 2024-06-28

**Authors:** George Paltoglou, Nickolas Ziakas, George P. Chrousos, Christos Yapijakis

**Affiliations:** 1 Unit of Endocrinology, Metabolism and Diabetes, First Department of Pediatrics, School of Medicine, National and Kapodistrian University of Athens, “Aghia Sophia” Children’s Hospital, 11527 Athens, Greece; gpaltoglou@med.uoa.gr; 2 Unit of Orofacial Genetics, First Department of Pediatrics, School of Medicine, National and Kapodistrian University of Athens, “Aghia Sophia” Children’s Hospital, 11527 Athens, Greece; nickolas_ziakas@hotmail.com; 3 University Research Institute of Maternal and Child Health and Precision Medicine, School of Medicine, National Kapodistrian University of Athens, 11527 Athens, Greece; chrousge@med.uoa.gr

**Keywords:** short stature, genetics, genetic syndromes, craniofacial morphology, cephalometric radiograph, growth hormone/insulin-like growth factor 1 axis, epiphyseal growth plate, cartilage extracellular matrix

## Abstract

**Introduction:** A plethora of biological molecules regulate chondrogenesis in the epiphyseal growth plate. Disruptions of the quantity and function of these molecules can manifest clinically as stature abnormalities of various etiologies. Traditionally, the growth hormone/insulin-like growth factor 1 (IGF1) axis represents the etiological centre of final stature attainment. Of note, little is known about the molecular events that dominate the growth of the craniofacial complex and its correlation with somatic stature. **Aim:** Given the paucity of relevant data, this review discusses available information regarding potential applications of lateral cephalometric radiography as a potential clinical indicator of genetic short stature in children. **Materials and Methods:** A literature search was conducted in the PubMed electronic database using the keywords: cephalometric analysis and short stature; cephalometric analysis and achondroplasia; cephalometric analysis and hypochondroplasia; cephalometric analysis and skeletal abnormalities; cephalometr* and SHOX; cephalometr* and CNP; cephalometr* and ACAN; cephalometr* and CNVs; cephalometr* and IHH; cephalometr* and FGFR3; cephalometr* and Noonan syndrome; cephalometr* and “Turner syndrome”; cephalometr* and achondroplasia. **Results:** In individuals with genetic syndromes causing short stature, linear growth of the craniofacial complex is confined, following the pattern of somatic short stature regardless of its aetiology. The angular and linear cephalometric measurements differ from the measurements of the average normal individuals and are suggestive of a posterior placement of the jaws and a vertical growth pattern of the face. **Conclusions:** The greater part of the existing literature regarding cephalometric measurements in short-statured children with genetic syndromes provides qualitative data. Furthermore, cephalometric data for individuals affected with specific rare genetic conditions causing short stature should be the focus of future studies. These quantitative data are required to potentially establish cut-off values for reference for genetic testing based on craniofacial phenotypes.

## 1. Introduction

Growth failure is a clinical entity comprised of two constituents, namely short stature (quantitative linear body growth) and/or growth faltering (linear body growth rate) [1]. Short stature in childhood and adolescence represents the most common reason for referral to paediatric endocrinology outpatient clinics [2,3,4]. Short stature is commonly defined to be a height that is less than two standard deviations below the average for that specific age, gender, and population, and, on standard growth charts, a drop below the third centile. The American Academy of Pediatrics recommends plotting the data on the World Health Organization charts for children aged less than two years and the Centers for Disease Control and Prevention’s 2000 charts for children aged two years or older [5,6]. For the Greek population, the most widely utilized charts are the Chiotis et al., 2003 growth charts [7].

In addition to the above definition, a child may be within the normal height range according to the height charts and still be considered as having idiopathic short stature if she or he is much shorter than their parents. 

A short-statured child can fall into one of three categories, which are useful for clinical purposes, namely primary growth failure, secondary growth failure, and idiopathic short stature [8]. Primary growth failure is of genetic origin, as one or more genetic defects cause(s) disruption of the events taking place at the epiphyseal growth plate, directly impeding its function. Secondary growth failure is associated with aetiologies extrinsic to the growth plate, including endocrine, paracrine, or nutritional factors, physical mechanisms, extracellular fluid parameters, proinflammatory cytokines and extracellular matrix molecules. Diagnosis of idiopathic short stature (ISS) is set by exclusion of the former two categories, therefore short stature with no recognizable genetic, endocrine, paracrine, nutritional, or other disorder.

Children with ISS have normal birth size and normal body proportions. ISS can either be asymptomatic or symptomatic, when there are one or more symptoms present, as revealed by clinical examination or medical history [6]. Additionally, ISS is further divided into familial and non-familial (sporadic), and each category can be characterized by normal or delayed puberty. In familial ISS, the child and one or both of the parents are below 2 standard deviations (SD) and the child’s curve runs close and parallel to the parents’ curve. In sporadic ISS both parents are of normal stature, but the child is below 2SD or under the target height. Treatment of children with ISS with recombinant human growth hormone (rhGH), albeit controversial, improves linear growth and adult height; a height difference of 5.3 cm in males and 4.7 cm in females was noted between a study group and a control group [2].

## 2. The Multifactorial Aetiology of Short Stature

Epiphyseal growth plate (EGP) is the key biological structure responsible for long bone elongation through the induction of chondrogenesis. This biological process determines the standing adult height. For many years, it was assumed that the sole mechanism regulating chondrogenesis at the EGP was through mesenchymal cell differentiation to chondrocytes and later chondrocyte maturation and proliferation, a process regulated by the human growth hormone/insulin-like growth factor 1 axis. However, advances in the fields of molecular biology and medical genetics have overturned this tenet and shed light on the biological factors orchestrating events at the EGP. Chondrogenesis in the EGP is strictly regulated by many intrinsic (endogenous to the growth plate) and extrinsic (exogenous to the growth plate) biological factors, which act either as activators or as inhibitors of chondrocyte metabolism and growth. These factors include endocrine, paracrine, or nutritional factors, physical mechanisms, extracellular fluid parameters, proinflammatory cytokines, extracellular matrix molecules and intracellular mechanisms. Genetic defects affecting one of the above mechanisms can severely disrupt the events taking place at the EGP during chondrogenesis and cause stature abnormalities.

**Figure 1:**
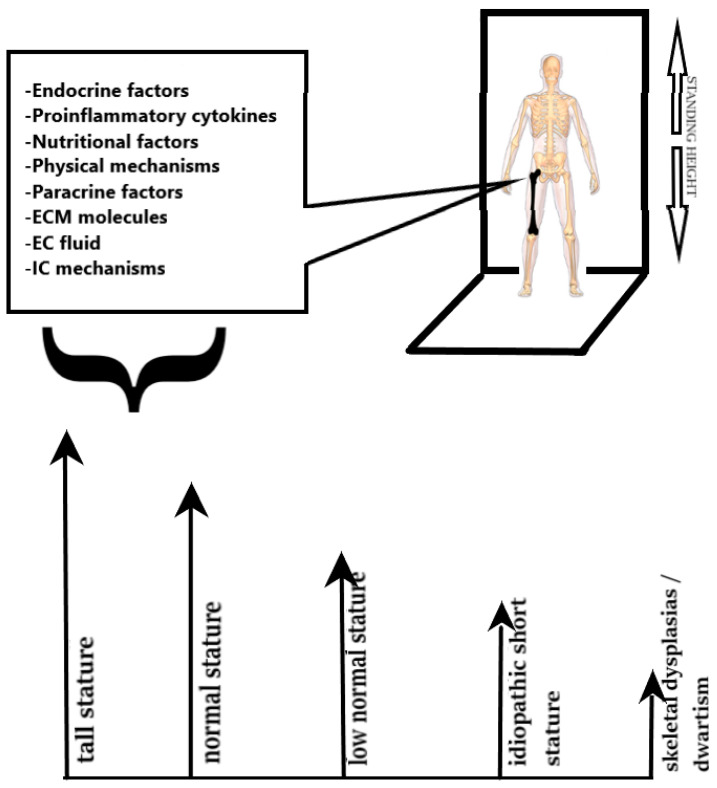
Factors affecting homeostasis of the epiphyseal growth plate and phenotypic manifestations of stature types resulting from complex interactions among these factors.

Stature is a multifactorial phenotypic trait influenced by multiple genetic and environmental factors. The genetic component is vast, as about 420 genetic loci contribute to adult stature, by encoding mainly proteins expressed in the EGP [9]. Some single gene defects can result in severe growth disorders, while most mutations or polymorphisms in various genetic loci cause low normal stature or idiopathic short stature. This observation highlights the fact that some of the genes involved play a crucial role in human growth, while others play a minor role. In addition, sequence variants in the same genetic locus can be expressed as different clinical entities. The phenotypic manifestation is the net result of the gene involved and the type of mutation in the gene (gain of function, altered function, impaired function or complete loss of function). Therefore, extreme tall stature, low normal stature, idiopathic short stature, and short stature are different phenotypic manifestations of the same clinical spectrum of disorders labelled under the term stature abnormalities (Figure 1). This approach to stature abnormalities has dictated the classification of short stature proposed by the European Society for Pediatric Endocrinology based on the causes of short stature into primary (of genetic origin) and secondary (of endocrine or other origin). This classification is less centred around pathophysiology and constitutes a more useful approach for clinical purposes [8,10,11,12,13,14].

## 3. Clinical Assessment of Children with Short Stature

Currently, there is no widely accepted diagnostic algorithm when assessing children with short stature referred to paediatric clinics. The clinician usually obtains a thorough medical history, including chief complaint (commonly being the short stature), history of the presenting complaint, past medical history, family history and pedigree, review of systems, and then carries out a thorough clinical examination. Meticulous measurements of height, body weight, head circumference, sitting height, arm span, body proportions (such as sitting height/height ratio, arm span/height ratio and upper arm/lower arm ratio), and pubertal status (rated according to Tanner) [1] are of paramount importance, as there might exist subtle disproportions that go by unnoticed even to the most experienced clinician. In addition, assessment of fontanelles, dentition status and any apparent dysmorphic features should be conducted. Radiologic imaging of the left hand and wrist is necessary for bone age assessment and evaluation of any incidental pathologic morphologic findings. Additionally, a blood sample is usually collected for preliminary biochemical assessment.

Clues collected from biochemical testing may lead the paediatrician to classify the possible aetiology of short stature into primary (genetic) or secondary. There are no clear guidelines as to when genetic testing must be addressed, but it relies heavily upon the clinical suspicion of the physician based on the findings from the physical, radiologic, and laboratory evaluation of the patient. Findings that raise suspicion for short stature of genetic aetiology include developmental delay, intellectual disability, behavioural problems, one apparently affected parent (with special attention on body proportions and dysmorphic features), family members with early onset arthritis or discopathy, microcephaly, relative macrocephaly, disproportionate ratios, extreme short stature, or apparent dysmorphic features. The growth chart can also provide valuable indications about a possible genetic aetiology, especially if there is a growth curve starting with a low or low-normal birth length, decreasing length SD for two to three years, followed by a stable height SD in childhood and a further decrease of height SD during adolescence. The genetic aetiology of stature abnormalities may be further elucidated in the future as more genetic tools are added to our diagnostic toolkit. For specific conditions, such as Noonan syndrome, scoring systems have already been proposed [15].

## 4. Cephalometric Assessment of Children with Short Stature

Cephalometric radiography is an extra-oral radiographic technique used to display the craniofacial complex in standardized repeatable conditions. The cephalometric radiologic system contains a special mechanical part called the cephalostat, which bears a plug inserted into the external ear canal, stabilizing the head and ensuring a constantly predetermined head position in a three-dimensional (3D) space. This results in a radiograph captured under standardized conditions that can be repeated with precision when it is required, because differences between radiographs should be real and not the result of different capture conditions. This technique offers the opportunity to conduct comparisons between radiographs of the same individual taken during different times or between radiographs of different individuals.

The cephalometric radiograph is part of daily dental and orofacial practice, especially in oral and maxillofacial surgery and orthodontics. It is used extensively in pre-treatment planning for assessing the quantitative extent of the existing oral and maxillofacial structure abnormalities and for verifying the clinical diagnosis, as well as post-treatment for the evaluation of the therapeutic result. There are two types of cephalometric X-rays, namely the lateral cephalometric X-ray and the frontal or postero-anterior cephalometric X-ray, based on the direction in which the X-ray beam is headed towards the face. Discrepancies affecting the structures of the craniofacial complex can be classified into three main categories as regards the skeletal plane to which they are orientated: anomalies in the anteroposterior (sagittal) plane, anomalies in the vertical (frontal) plane, and anomalies in the transverse plane. Most of the existing abnormalities concern the first two planes and the lateral cephalometric X-ray. The use of the frontal cephalometric X-ray is very limited due to the extensive structural noise that the radiologic image contains, but it is the only technique that can assess abnormalities in the transverse plane, such as a posterior cross-bite or a facial asymmetry.

**Figure 2:**
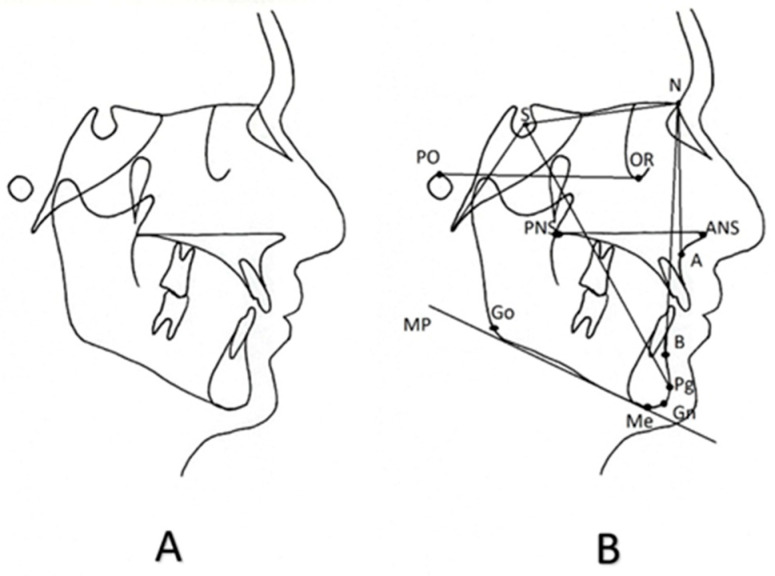
(**A**) Lateral cephalometric tracing, (**B**) cephalometric landmarks and the lines connecting them that are referred to as cephalometric reference planes. The most utilized cephalometric landmarks and planes are depicted. PO: porion; S: sella; N: nasion; OR: orbitale; PNS: posterior nasal spine; ANS: anterior nasal spine; A: A-point, B: B-point, Go: gonion, Pg: pogonion, Gn: gnathion, Me: menton; SN: Anterior cranial base plane; sBa: Posterior cranial base plane; PoOr: Frankfurt horizontal plane; PNS-ANS: Palatal plane; GoMe: Mandibular plane; *Y*-axis SPg or SGn; NA plane; NB plane; NPg: Facial plane.

To provide diagnostic information, the cephalometric radiograph undergoes a process called “tracing”. This can be done either by hand or digitally with the use of applications on a computer. Specific bony or soft tissue points on the cephalometric tracing are identified and marked as cephalometric landmarks. The lines connecting the cephalometric landmarks are called “cephalometric reference planes”, despite being two-dimensional structures. Finally, various linear, angular, or even radial measurements are conducted between the cephalometric landmarks and cephalometric planes. This process constitutes the cephalometric analysis. Figure 2 depicts lateral cephalometric tracing conducted by hand on transparent paper and indicates the most common cephalometric landmarks and reference planes.

## 5. Cephalometric Data for Genetic Syndromes Associated with Short Stature

There are several studies assessing specific genetic syndromes that present with short stature. To review the literature, a search was conducted in the PubMed electronic database using the following search criteria: cephalometric analysis and short stature; cephalometric analysis and achondroplasia; cephalometric analysis and hypochondroplasia; cephalometric analysis and skeletal abnormalities; cephalometr* and SHOX; cephalometr* and CNP; cephalometr* and ACAN; cephalometr* and CNVs; cephalometr* and IHH; cephalometr* and FGFR3; cephalometr* and Noonan syndrome; cephalometr* and Turner syndrome; cephalometr* and achondroplasia. Extensive cephalometric analysis studies have been conducted for common genetic syndromes, such as achondroplasia, Turner syndrome, and Noonan syndrome. Cephalometric data for rarer genetic syndromes are scarce. Articles that could not be accessed online as well as articles that were not written in English were excluded, while all the other articles were included in the study. Table 1 summarizes cephalometric data for common genetic syndromes associated with short stature.

### 5.1. Achondroplasia (OMIM 100800)

Achondroplasia is a rare genetic disorder with an estimated birth prevalence of about 4 per 100,000 live births worldwide. Achondroplasia is caused by mutations (G1138A and G1138C) in the fibroblast growth factor receptor 3 (FGFR3) gene located on chromosome 4p16.3. It is an autosomal dominant disorder, but about 80% of cases are sporadic due to a de novo mutation in the offspring of unaffected parents [16]. Lateral cephalometric radiographs of individuals with achondroplasia demonstrate:

(a) Skull: enlarged skull calvaria, frontal bossing, high degree of pneumatization of the frontal sinuses, acute occipital prominence, and a short, deformed, and depressed nasal bone. Normal length of the anterior cranial base, remarkably reduced length of the posterior cranial base and acute cranial base angle [17,18,19,20]. The length of the cribriform plate of the ethmoidal bone is remarkably shortened in contrast to the anterior sphenoidal length, which is remarkably augmented, possibly counterbalancing the underdeveloped cribriform plate, and suggesting that growth in the anterior cranial base takes place primarily at the spheno-ethmoidal synchondrosis. The reduced posterior cranial base length possibly results from the hypo-development of the cartilaginous bone with possible premature ossification of the spheno-occipital synchondrosis. The exaggerated reduction of the cranial base angle in achondroplasia may be related to a larger brain and possibly premature ossification of the intersphenoid synchondrosis [18].

(b) Anteroposterior plane: Posteriorly placed and smaller maxilla, anteriorly or normally placed mandible of normal size with a normal gonial angle, anteroposterior arrangement of the jaws, expressed as a skeletal class III pattern of malocclusion resulting in anterior cross-bite [17,18,19,20].

(c) Vertical plane: Reduced upper anterior facial height, posterior tilt of the nasal floor (palatal plane), and high coronoid process [17,18,19].

(d) Teeth and soft tissue: Maxillary incisors proclined relative to the Frankfurt horizontal plane and mandibular incisor angulations normal relative to the mandibular plane [19].

Lateral cephalometric radiographs of achondroplastic transgenic mice expressing a constantly active mutant of MEK1 in chondrocytes revealed significantly reduced skull length but significantly increased cranial arch length. The area of the braincase was significantly larger and more circular. The maximum diameter was normal, but the minimum diameter, roughly equivalent to braincase height, was increased [21].

### 5.2. Turner Syndrome (OMIM 309585)

Turner syndrome is a common chromosomal aberration caused by a complete or partial loss of one copy of the X chromosome (45,X) or by mosaicism of a 45,X cell line with a normal 46,XX cell line with an incidence of up to 1 per 2000 live-born girls [22]. The 45,X karyotype is estimated to be present in 3% of all conceptions, but almost 99% of these abnormal foetuses spontaneously abort, usually during the first trimester of the pregnancy, accounting for up to 10% of all spontaneous abortions [22]. The cephalometric data available for individuals with Turner syndrome include:

(a) Skull: Posterior cranial base exhibiting reduced length and cranial base angle being increased (increased angle of flexion), causing a flattening of the cranial base [23,24,25,26,27,28,29,30,31,32,33,34,35,36,37,38,39], smaller dimensions of the calvarium of the skull [35,37], reduced thickness of the calvarium [39], fused cervical vertebrae [40], a more inferiorly and anteriorly placed external acoustic meatus [41], smaller and more delicate mastoid processes and of reduced pneumatization, larger and excessively pneumatized sphenoidal sinuses, smaller Sella turcica [42], premature calcification of the petro-clinoid ligament in patients before the age of 20 [39], small facial part of the skull compared with the cerebral part [39], retarded development of the skull affecting cranial growth as well as growth in the condyle and the spheno-occipital synchondrosis [43]. Most prominent discrepancies from normal controls are observed in 45,X individuals, while a milder phenotype is noted in individuals bearing an isochromosome and even milder differences in mosaic 45,X/46,XX individuals [24,25,35].

(b) Antero-posterior plane: Full Turner individuals (45,X) exhibit the most remarkable aberrations of craniofacial morphology [24,25,35], including reduced length of the maxilla [24,25,26,29,30,32,33,35,42,44,45,46,47,48], reduced length of the mandible [25,26,27,30,31,32,34,35,36,37,42,45,46,47], posteriorly positioned maxilla [24,25,26,27,28,34,35,37,42,44,45,46,49,50,51], posteriorly positioned mandible in relation to the anterior cranial base [24,25,26,27,28,33,35,37,42,44,45,46,49,50,51], and posteriorly positioned chin [49]. Cases of normal maxillary [34,36] and mandibular length [44] have also been reported. In fact, Rzymski et al. have reported a disproportionately large mandible in females with Turner syndrome, as if it did not fit in with the remaining facial bones, that were squared with pronounced angles, resembling a man’s jaw [39]. The above arrangement of the jaw bones leads to a skeletal class II pattern in most individuals with Turner syndrome [32,45,46,47]. However, a skeletal class III pattern has also been reported, with both the maxilla and the mandible being posteriorly positioned [50]. Growth hormone has a beneficial effect on maxillary and mandibular growth, as shown by such treatment of individuals with Turner syndrome who have demonstrated a class I skeletal pattern [52,53,54].

(c) Vertical plane: The cephalometric angles assessing the anomalies in the vertical plane (MP-SN, MP-FH, MP-PP) seem to be increased in individuals with Turner syndrome, constituting the skeletal planes as hyperdivergent, and implying a posterior/downward inclination of the mandible with a vertical growth pattern of the face [24,25,26,31,33,34,35,37,40,43,45,49,50,55], a reduction of the posterior facial height, and an increase in the anterior facial height. Other findings include the presence of a deep bite resulting from the increased vertical overjet [37,46], a shortened distance of glenoid fossa and gonion from the Sella [26], as well as an anterior open bite [47]. Individuals with Turner syndrome treated with growth hormone demonstrated normalization of the vertical characteristics after the treatment [52,53,54], while posterior facial height, mandibular ramus height, and anterior facial height were mostly influenced [53].

(d) Coronal plane: Individuals with Turner syndrome exhibited facial asymmetry [40,45] and posterior cross-bite as a result of the transversal dimension reduction of the maxilla [32,47].

(e) Teeth and soft tissue: The occlusal plane angle is remarkably tilted, the maxillary incisors are lingually inclined, and the upper and lower lips are 1 mm and 5 mm forward of the E line [50]. In addition, both the maxillary and the mandibular incisors reportedly exhibit normal inclination and position in relation to their skeletal base [44], the teeth roots are short [47], the position of the tongue is remarkably low [56], and the pharyngeal airway space is narrower in all its dimensions [27,46].

### 5.3. Noonan Syndrome

Noonan syndrome is characterized by genotypic and phenotypic variance, which makes it difficult to diagnose individuals with milder phenotypes. It is a common genetic abnormality with an incidence of one in 1000–2500 live births for the severe phenotype, but milder phenotypes emerge up to ten times more frequently. Familial recurrence is consistent with an autosomal dominant mode of inheritance, but de novo mutations are more common and encountered in the majority of individuals [57]. There are also at least two autosomal recessive forms of the syndrome: NS2 (OMIM 605275) and NS14 (OMIM 619745). The cephalometric data available for individuals with Noonan syndrome include the following:

(a) Skull: In subjects with Noonan syndrome, the distance by which the odontoid tip extended past McGregor’s line is significantly greater than that of the control subjects; the third and fourth cervical vertebrae are in significantly superior positions and also significantly superior to the hyoid bone in subjects with Noonan syndrome compared to controls. There is no difference in the position of the hyoid bone between the groups [58]. The head circumference of all females with Noonan syndrome is normal, whereas in 50% of males, it is below the 3rd percentile for age [59].

(b) Anteroposterior plane: Skeletal abnormalities in the anteroposterior plane do not seem to demonstrate a specific pattern in individuals with Noonan syndrome. However, the length of the jaws seems to be reduced. There are reports of class I malocclusion [60,61,62]. Several reports indicate a posteriorly placed mandible [15,63,64,65].

(c) Vertical plane: Most reports indicate a downwards mandibular growth pattern with hyperdivergent planes and increased vertical angles [15,62,63,64,66]. Nevertheless, cases of a horizontal growth pattern [60], an increased vertical overjet [62,63], and a decreased vertical overjet [61,65] have been reported.

(d) Teeth and soft tissue: The external ear position in individuals with Noonan syndrome does not differ significantly from that of healthy controls [41]. The mandibular incisors seem to be labially inclined in most reports [61,64], but not all of them [60,62].

### 5.4. Idiopathic Growth Hormone Deficiency (262400 Type IA; 612781 Type IB; 173100 Type II; 307200 Type III; 618157 Type IV)

All linear measurements were reduced in a study of isolated growth hormone deficiency (IGHD) individuals [67]. Maxillary length was the most affected measurement, followed by posterior cranial base length, total mandibular length, total posterior facial height, total anterior facial height, mandibular corpus length, and anterior cranial base length. Angular measurements were in the normal range, except for increased gonial angle. Posterior facial height/anterior facial height and lower-anterior facial height/anterior facial height ratios were not different from those of the control group [67].

Growth hormone treatment had a reportedly favourable influence on the craniofacial growth pattern of 21 boys with idiopathic short stature (ISS) and 25 boys with growth hormone deficiency (GHD). Before human growth hormone treatment, all the craniofacial structures had reduced length, as well as a retrognathic facial type and a skeletal class II tendency [68]. After treatment, the jaw and the cranial base angles were increased in children with short stature, while the angles between the anterior cranial base and the mandibular plane and between the maxilla and mandible were larger than normal. The proportions between anterior and posterior face heights and between upper and lower anterior face heights were also smaller [68]. During the treatment period, an overall enhancement in growth of the facial skeleton was observed in boys with short stature. The changes induced by growth hormone yielded a more prognathic growth pattern, a more anterior position of the jaws in relation to the cranial base and increased anterior rotation of the mandible.

Both the length of the mandible and the anterior facial height were greater in boys who received growth hormones. No differences were noted between the GHD and ISS boys. The ramus height displayed a greater change in the individuals who had not received orthodontic treatment. Another study noted that in individuals with idiopathic growth hormone deficiency who did not receive growth hormone treatment, the anterior cranial base length, anterior facial height, maxillary length, mandibular length, and ramus height were smaller, while treated individuals had a significantly larger upper anterior facial height, maxillary length, and ramus height [69].

### 5.5. Prader–Willi Syndrome (OMIM 176270)

Among 42 individuals with Prader–Willi syndrome (PWS), 12 demonstrated a posteriorly placed mandible with a skeletal class II pattern, vertical growth direction, and increased anterior facial height, 10 individuals had reduced cranial base angle, a skeletal class II pattern and an increased lower anterior facial height, while 20 had an anteriorly placed mandible, a skeletal class III pattern with anterior growth direction, lingually inclined mandibular incisors, and labially inclined maxillary incisors [70].

In another report of 20 subjects with PWS, the length of the maxilla, the length of the mandible at both the ramus and the mandibular body, and the posterior and anterior facial heights were significantly reduced, while the angular measurements did not differ significantly from normal values. The anteroposterior cephalometric radiographs revealed a significant reduction in maxillary skeletal width, mandibular skeletal width, and interzygomatic distance [71]. Similar findings were noted in most individuals of another cohort of PWS patients. The mandibular and maxillary length, ramus height, posterior facial height, and mid-facial height were all significantly smaller, while the overall small bony structures in contrast to the relatively large soft tissue draping were observed especially in obese adults. The data suggest that a characteristic bony model might be created for PWS, which could be of use both in the diagnosis and treatment of such patients by orthodontists [72]. A positive correlation between cephalometric measurements, gender, crown length of permanent left central incisor and combined mesiodistal diameter of permanent maxillary anterior with somatic stature was found in 70 individuals with PWS [73].

### 5.6. Down Syndrome (OMIM 190685)

Individuals with Down syndrome show reduced anterior cranial base length with an increased cranial base angle, reduced mandibular length and upper anterior and lower anterior facial height with a normal posterior facial height [74]. Maxillary length and facial convexity were reduced compared with the control group with a class I skeletal pattern, although the anteroposterior position of the maxilla was normal. Individuals with Down syndrome had a reduced maxillary length with a flatter cranial base and thus differed from the typical class III skeletal pattern. Maxillary deficiency was not prominent in the face of individuals with Down syndrome because of the overall reduction in craniofacial dimensions [74]. A report of children with Down syndrome with and without hypodontia indicated that individuals with hypodontia presented over the years a reduced ANB angle and a decrease in vertical angles compared to subjects without hypodontia [75]. In another report of Down syndrome patients, the cranial base angle was normal, and the length of the anterior cranial base was reduced in 54% of the individuals, while the jaws presented a normal relationship in both the anteroposterior and the vertical planes [76]. An anterior cross-bite was observed in 38% and a reduced interincisal angle in 77% of the individuals. The lower incisors were anteriorly placed in 84% and were labially inclined in 77% of the individuals. The upper incisors were anteriorly placed in 77% of the samples, while the lower lip protruded in 84% of samples [76]. The most common abnormality observed in Down syndrome patients from Pakistan was a fusion between the second and third cervical vertebrae. This abnormality was found in 1/5 of subjects with skeletal class I and 1/2 of subjects with skeletal class II and III patterns [77]. The highest frequencies of partial cleft at the height of the first cervical vertebra and occipitalization were noted in individuals with skeletal class II and III patterns, respectively; however, no individual displayed fusion between the second and third cervical vertebrae or dehiscence. Furthermore, individuals with skeletal class III presented more frequent abnormalities of the cervical vertebrae in contrast to individuals with skeletal class I malocclusions [77].

### 5.7. Muenke Syndrome (OMIM 602849)

Muenke craniosynostosis or coronal synostosis syndrome is caused by a point mutation (C749G) in the FGFR3 gene resulting in a Pro250Arg (P250R) substitution [78]. The cephalometric data for these patients include:

(a) Skull: Decreased intracranial volume, significantly reduced length of the skull length and significant reduction of the anterior cranial base length [78,79,80]. The angle between the cranial base and the Frankfurt horizontal plane is increased [78]. The anterior part of the skull is characterized by a significant increase in the intercoronal distance. Bilateral interorbital and anterior interorbital distances are increased, confirming a hypertelorism typical for this syndrome. The “frontal bossing” frequently found in brachycephaly is characterized by increased sagittal extension of the forehead and by the increased height of the frontal prominence. Following surgery (fronto-orbital advancement), the measurements defining the morphology of the forehead are reduced and appear to stabilize during future examinations [80].

(b) Anteroposterior plane: Reduced length of the maxilla [79,80] and reduced length of the mandible [78,79]. The severely reduced maxillary length is held accountable for the midface deficiency in those individuals [80,81]. The maxilla is posteriorly placed, while the mandible is normally placed [82]. Most patients exhibit class I skeletal relation [82] but class II and III have also been described [82].

(c) Vertical plane: Reduced upper and lower anterior facial height [79], hyperdivergency of the skeletal planes, increased gonial angle, and an anterior open bite [78].

(d) Transverse plane: Increased facial width and significant skeletal asymmetry [79]. Prominent bulging of the temporal fossa is frequently responsible for poor morphological outcomes in carriers of the P250R mutation and it is corrected primarily along with the fronto-orbital advancement surgery [83].

### 5.8. Other Genetic Syndromes

Table 2 summarizes cephalometric data for less common syndromes associated with short stature.

**22q11.2 deletion syndrome (OMIM 611867)**: In 20 patients, the cranial base angle was increased and the mandible was posteriorly placed [84].

**49,XXXXY syndrome**: In a 9-year-old boy, the maxilla was placed in a normal anteroposterior position, the mandible was slightly anteriorly placed, and the mandibular incisors were lingually inclined [85].

**Acrofacial dysostosis—Catania brachydactylous type (OMIM 101805)**: Analysis of cephalometric and hand-wrist radiographs of a mother and daughter showed no distinctive diagnostic abnormalities [86].

**Beckwith–Wiedemann syndrome (OMIM 130650):** Most of the 25 children (76%) exhibited hyperdivergent skeletal planes with dental class I relation. About 40% had an anterior open bite and 16% also showed unilateral posterior cross-bite. No statistically significant differences were observed among the different phenotypes of Beckwith–Wiedemann syndrome [87].

**Bloch–Sulzberger syndrome (OMIM 308300)**: In the case of an 8-year-old female patient with cleft lip and palate, the length of the maxilla was reduced, the skeletal planes were hyperdivergent, and the maxillary incisors were lingually inclined [88].

**Chronic acid sphingomyelinase deficiency**: Four individuals had a posteriorly placed maxilla and one patient had normal anteroposterior maxillary positioning. Three individuals displayed a normal nasolabial angle, while it was increased in two individuals. The mandible was posteriorly placed in four patients and only one patient had an anteriorly placed mandible. The facial convexity was prominent, as well as the posteriorly placed maxilla and mandible and the skeletal class II pattern. Three individuals displayed normal positioning and two displayed retroinclination of the upper incisors. As for the mandibular incisors, three individuals displayed normal positioning, one displayed a reduced inclination, and one displayed an augmented inclination [89].

**Cockayne syndrome (OMIM 216400)**: Cephalometric analysis of nine patients revealed hypo-development of the skull, reduced length of the mandible, posteriorly placed and shorter mandible and skeletal class II pattern [90].

**Craniosynostosis, syndromic with fused or open spheno-occipital synchondrosis:** Patients with syndromic craniosynostosis usually have more severe symptomatology in comparison to patients with single suture synostosis [91]. The fused group of patients displayed a higher frequency of severe midface deficiency compared to the non-fused group (70% versus 10%). The fused group showed relatively higher percentages of severe class III skeletal pattern (100% vs. 70%), severely hyperdivergent skeletal planes (40% vs. 10%), severely forward condyle position (30% vs. 0%), and moderate and severe upward anterior cranial base inclination (90% vs. 50%) than the open group. However, the two groups displayed the same distribution of moderately and severely retrusive orbital positions [92].

**Εctodermal dysplasia 1, hypohidrotic, X-linked (OMIM 305100)**: In 48 patients, the maxilla exhibited reduced length and was posteriorly placed. The length of the mandible was normal, but it was anteriorly placed with a protruding chin. The skeletal pattern was class III, and the anterior facial height was reduced. Male patients with numerically more clinical manifestations of ectodermal dysplasia exhibited a larger reduction in the length of the maxilla and a more posteriorly placed maxilla, as well as larger vertical angles and an increased anterior facial height, in comparison with male patients bearing fewer manifestations of ectodermal dysplasia [93]. In another report, six patients exhibited a reduced length of the maxilla and an anteriorly placed mandible. Hyperdivergent skeletal planes, reduced anterior facial height, and reduced upper anterior facial height were also present. The first maxillary molars were located in higher positions suggesting that the vertical and anteroposterior maxillary growth retardation, rather than the lack of firm interdental contacts due to the lack of numerous teeth, is the cause of the reduced anterior facial height in individuals with ectodermal dysplasia [94].

**Ectodermal dysplasia, anhidrotic, with cleft lip/palate or Rapp-Hodgkin syndrome (OMIM 129400):** In three patients in a family with a rare form of ectodermal dysplasia, the length of the maxilla was mildly to moderately reduced. The maxilla was placed closer to the anterior cranial base, but it was positioned within normal limits anterior-posteriorly relative to the forehead (Lande’s angle). The presence of an anteriorly placed mandible also contributed to a concave skeletal profile [95].

**Ellis–Van Creveld syndrome (OMIM 225500)**: Among seven individuals, two demonstrated a class I skeletal pattern, four demonstrated a class III pattern (due to anteriorly placed mandible in two patients and posteriorly placed maxilla in two patients), and one individual demonstrated a class II pattern due to the posteriorly placed mandible. Regarding the vertical plane, four individuals showed a hyperdivergency of the skeletal planes, two individuals were in the normal range, and one individual showed a horizontal growth pattern. Incisor retroclination was profound in the upper and lower teeth in four individuals, while soft tissue analysis indicated upper lip retrusion in four individuals and lower lip retrusion in one individual. One individual displayed a convex profile, whereas two patients exhibited concave profiles [96]. A high degree of agreement was observed between the two methods for determining skeletal age (degree of cervical vertebral maturation in lateral cephalometric radiography and hand-wrist method) in 178 patients with short stature, especially in girls in the familial short-stature group, whereas boys had higher agreement in the nonfamilial short-stature group [97].

**Hajdu–Cheney syndrome (OMIM 102500)**: In the case of a 22-year-old female, cephalometric analysis revealed anteroposterior and vertical hypoplasia of the midface. The cranial base was also increased. The maxilla and the mandible were placed posteriorly, although their relationship was within normal limits. The Sella turcica was enlarged, elongated, and wide open with slender clinoids [98].

**Hallermann–Streiff syndrome (OMIM 234100)**: In a 10-year-old girl, the mandible had reduced length and was posteriorly placed, resulting in a skeletal and dental class II pattern. In the vertical plane, the patient presented a vertical growth pattern with an opening of the gonial angle, a large anterior open bite, and an excessive increase in the lower anterior facial height [99].

**Hemifacial microsomia (OMIM 141400):** In 183 patients, the ratios of the microsomic side to the normal side of the lateral distance of the condyle, the mandibular ramus height, and the length of the body of the mandible were significantly reduced. The inclination of the body of the mandible was greatly augmented on the side with hemifacial microsomia than on the normal side, and the extent of the mandibular ramus was significantly lower than on the normal side. The affected/unaffected ratios of the extent of the angle of the mandible and the inclination of the body of the mandible were increased. Moreover, the length and the inclination of the body of the mandible had significant correlations with the distance of the shift of the menton [100].

**Kabuki syndrome (OMIM 147920):** A male patient had a posteriorly placed maxilla and mandible with a skeletal class I pattern, increased lower anterior facial height and a severe anterior open bite. During the follow-up, the anteroposterior growth of the maxilla and mandible showed improvement. The skeletal pattern changed to skeletal class III from skeletal class I during the follow-up period. Both the upper and the lower anterior facial heights increased [101].

**Klippel–Feil syndrome (OMIM 616549):** Cephalometric analysis of a case of an 8-year-old female revealed a class I skeletal pattern with a vertical growth pattern and fused cervical vertebrae [102]. A case of a 12-year-old female with Turner syndrome combined with Klippel–Feil syndrome demonstrated a skeletal class III pattern [40].

**Langer–Giedion syndrome (OMIM 190350)**: A 10-year-old child had both a posteriorly placed maxilla and mandible [103].

**Larsen syndrome (OMIM 150250)**: In the case of a 5-year-old girl, both the maxilla and the mandible were posteriorly positioned, forming a skeletal class III pattern. The vertical angles were increased with an augmented angle of the gonion, and development of the lower face toward the postero-inferior direction. The inclination of the upper incisors was within the normal range, while the lower incisor was more lingually positioned. The orbits were also positioned posteriorly relative to the anterior cranial base. The anteroposterior cephalometric radiograph revealed hypertelorism and a narrow basal part of the maxilla, as well as narrower upper and lower dental arches [104].

**Methylmalonic aciduria** and **homocystinuria (OMIM 277410):** An 11-year-old patient who developed the disorder during the neonatal period exhibited a postural alteration in which the head was rotated and bent towards the left shoulder, which was lower than the right one. That abnormality was responsible for disturbing muscular and skeletal equilibrium in the frontal plane, thus causing the horizontal planes of both maxillary bones to converge towards the right, as indicated by the postero-anterior cephalogram [105].

**Moebius syndrome (OMIM 157900)**: Among 24 patients with classic and incomplete types, about 2/3 presented either small or posteriorly placed jawbones, almost all displayed mandibular hypoplasia, and 3/4 had a skeletal class II pattern. Maxillary height was increased, resulting in a vertical growth pattern. Upper and lower incisors exhibited proclination, the upper and lower lips protruded, and a long upper lip was evidenced in 41% of the patients. No statistically significant differences were noted when comparing classic and incomplete Moebius syndrome [106].

**Myotonic dystrophy type I (OMIM 160900), congenital or childhood-onset**: Larger ANB angle values and smaller facial angle values were observed in 15 patients. In the vertical plane, the skeletal planes were hyperdivergent with mandibular plane angle and the intermaxillary angle increased. During a 5-year follow-up period, the intermaxillary angle remained the same in individuals with myotonic dystrophy type I, whereas this angle decreased in healthy individuals [107].

**Orofaciodigital syndrome type I,** also called **Papillon-Léage-Psaume syndrome (OMIM 311200)**: Two sisters had an apparent dolichocephaly and leptoprosopic face, a skeletal open bite, and a bimaxillary retrognathism [108].

**Rubinstein–Taybi syndrome (OMIM 180849):** Most of the eight individuals demonstrated a skeletal class II pattern and brachycephaly [109].

**Seckel syndrome (OMIM 210600)**: Four siblings (two girls and two boys) had remarkably small skulls with an extremely short anterior cranial base and maxillary length. Differences in the morphology of the Sella turcica were observed between girls and boys [110].

**Silver–Russell syndrome (OMIM 180860)**: A skeletal class II pattern was observed in 18 individuals, a class I pattern was observed in 3 patients, and a class III pattern was observed in one individual, while almost all patients (n = 21/22) displayed a molar dental class II pattern. All the patients with a skeletal class II pattern had a posteriorly placed mandible with a reduced ramus length and short horizontal branch. Among the patients with the class I pattern, two presented a reduced length of the maxilla and a posteriorly positioned mandible, and one patient presented anterior placement of both the maxilla and the mandible. The sole patient with a skeletal class III pattern showed a shorter and posteriorly placed mandible associated with a shorter maxilla. The hyoid bone was in a normal position in 15 cases, accessioned (from 2–4 mm up) in 4 cases, and lowered (from 1–9 mm down) in 3 cases [111].

**Simpson–Golabi–Behmel syndrome (OMIM 312870)**: A 6-year-old boy and an 8-year-old boy exhibited increased length of the anterior cranial base, and increased length of the maxilla and the mandible [112,113]. The 6-year-old had a class I skeletal pattern and hyperdivergent skeletal planes with increased lower anterior facial height and increased gonial angle [112]. The 8-year-old boy had a skeletal class III pattern, and an increased lower anterior facial height [113].

**Solitary median maxillary central incisor syndrome (OMIM 147250):** In the case of an 11-year-old girl with panhypopituitarism, a hypoplastic Sella turcica was observed in addition to a skeletal class III pattern with a convex profile, as a result of reduced maxillary length and anteriorly placed mandible. Other findings included hyperdivergency of the skeletal planes, cervical vertebral maturation at cervical stage 2, airway patency, anterior crossbite, and upper and lower incisal proclination. No family history of skeletal class III pattern was reported [114].

**Treacher–Collins syndrome (OMIM 154500):** Reduced length of both the anterior and posterior cranial base and reduced cranial base angle was observed in 20 patients. The maxilla was posteriorly placed and exhibited reduced length. The mandible was also posteriorly placed with a characteristic reduction of the mandibular length and decreased maximum ramus width. The skeletal planes were hyperdivergent and the gonial angle was increased. Both the anterior and posterior facial heights were decreased, although the lower anterior facial height was increased. The maxillary and functional occlusal planes were tipped upwards posteriorly [115].

**Williams–Beuren syndrome,** also called **Williams syndrome (OMIM 194050):** Eight patients had reduced length of the anterior cranial base, although the cranial base angle was normal. The chin was posteriorly placed, although the mandible had a normal position in the anteroposterior plane. The skeletal planes were hyperdivergent, although the total anterior facial height was normal. Despite the normal total anterior facial height, there was an unusual anterior facial height ratio and posterior-to-anterior facial height disproportion [116]. In 17 individuals, the most affected parameters were those associated with the mandibular incisors [117].

## 6. Cephalometric Data for Gene Variants Associated with Short Stature

There are several studies assessing specific pathogenic or likely pathogenic genetic variants associated with short stature. To review the literature, a search was conducted in the PubMed electronic database using the following search criteria: cephalometric analysis and short stature; cephalometric analysis and gene variant; cephalometr* and SHOX; cephalometr* and GHR. No studies were found providing cephalometric data for individuals with copy number variations (CNVs) or some variants in genes known to be associated with growth disorders [118]. For example, no studies were found providing cephalometric data for individuals with mutations in the Aggrecan (*ACAN*) gene located on 15q26, which causes spondyloepimetaphyseal dysplasia aggrecan type (OMIM 612813), spondyloepiphyseal dysplasia type Kimberley (OMIM 608361), and short stature with advanced bone age (OMIM 165800).

### 6.1. Growth Hormone Receptor Gene (GHR Gene)

The *GHR* gene (5p13.1–p12) codes for the growth hormone receptor protein. Mutations in this gene have been associated with Laron syndrome (OMIM 262500), also known as growth hormone insensitivity syndrome (GHIS), a disorder characterized by proportional dwarfism. Individuals carrying the CA genotype of the rs6184 single nucleotide polymorphism in the *GHR* gene showed both significantly decreased values for ANB angle (a tendency for class III skeletal pattern) and increased mandibular length. No statistically significant differences amongst genotype groups of rs6180 single nucleotide polymorphism were observed. Moreover, the CA genotype of rs6184 SNP and the AA haplotype were highly correlated with class III skeletal pattern [119].

### 6.2. Short Stature Homeobox Containing Gene (SHOX Gene)

The *SHOX* gene is located in pseudoautosomal region 1 on the distal end of the short arm of chromosomes Xp22.33 and Yp11.32. *SHOX* haploinsufficiency can phenotypically express as idiopathic short stature, Leri–Weill dyschondrosteosis (OMIM 127300), or Langer mesomelic dysplasia (OMIM 249700). In the case of a 24-year-old male with Leri–Weill dyschondrosteosis, the lateral cephalogram showed a posteriorly placed maxilla, a posteriorly placed mandible with reduced ramus and body length, an increased mandibular angle, and a class III skeletal relation with hyperdivergent skeletal planes [120]. A 17-year-old female with Leri–Weill dyschondrosteosis had an abnormal craniofacial morphology due to craniosynostosis through premature fusion of the squamosal sutures and partial closure of the coronal sutures. The skull calvarium was bulging through the partially synostosed coronal and totally synostosed squamosal sutures [121]. A 10-year-old male with Leri–Weill dyschondrosteosis and congenital conductive loss of hearing had frontal bossing, low nasal bridge, maxilla deficient in both the anteroposterior and vertical plane and a slightly anteriorly placed mandible, with a posteriorly placed chin, resulting in a mesio-relation of the jaws and a negative horizontal overjet of the incisors. The lower anterior facial height was normal [122].

### 6.3. Natriuretic Peptide Precursor C Gene (NPPC Gene)

*NPPC* gene resides on chromosome 2q24-qter. Mutations in the gene express as acromesomelic dysplasia Maroteaux type (OMIM 602875) or disproportionate short stature. Cephalometric data exist only for mice with *NPPC* gene mutations [123]. The craniofacial complex morphology in knockout mice not expressing the *NPPC* gene and transgenic mice overexpressing the *NPPC* gene were compared to that of wild-type mice. Three-dimensional reconstructions of microCT skull images demonstrated reduced skull lengths in the knockout mice. Nose length, nasal bone length, and maxilla length were significantly reduced. The mandible was also affected, exhibiting a shorter length, but the phenotypic aberration was milder. Transgenic mice exhibited increased cranial length with increased nose length, nasal bone length, and maxilla length, but their mandibular length did not differ from wild-type mice. Skull width and interorbital distances were normal. The nasal, premaxilla, maxilla, and frontal bones were more notably altered than the neurocranium, resulting in hypo-development in knockout mice skulls or hyper-development in transgenic mice skulls in the sagittal plane. Knockout mice showed mandibular hypoplasia at the centre of the condylar process and angular process in the sagittal direction. No difference was noted among the lower jaws of wild-type and transgenic mice. The skull base was shorter in knockout mice and longer in transgenic mice compared with wild-type mice. Both the occipital and sphenoid bones that comprise the skull base were significantly reduced in knockout mice and greater in transgenic mice than those of wild-type mice [123].

### 6.4. Indian Hedgehog Gene (IHH Gene)

Mutations in the *IHH* gene (2q35-36) are responsible for brachydactyly type A1 (OMIM 112500) and acrocapitofemoral dysplasia (OMIM 607778). When 50 female mice were fitted with orthodontic functional appliances placing their mandibles in a more anterior position, the mandibular advancement triggered *IHH* gene expression in condylar cartilage and increased the replicating mesenchymal cell population [124]. These findings suggest that the *IHH* gene acts as a mediator of mechanotransduction that converts mechanical signals resulting from anterior mandibular displacement to stimulate cellular proliferation in condylar cartilage.

### 6.5. Fibroblast Growth Factor Receptor 3 Gene (FGFR3 Gene)

The *FGFR3* gene (4p16.3) encodes the most significant receptor of fibroblast growth factor, a paracrine signal molecule acting directly upon the growth plate chondrocytes, by binding to its receptors on the chondrocyte cell membrane. FGFR3 bears an extracellular part responsible for binding the ligand and an intracellular part responsible for mediating the intracellular signal transduction through activating the Ras/MAPK pathway, which leads to chondrocyte cease of multiplication. The biological significance of this interlude is to give time to the newly differentiating osteoblasts to form new bone and for the biological processes of chondrogenesis and osteogenesis to take place in conjunction.

Mutations in the *FGFR3* gene manifest a perfect example of how different mutations in the same gene are expressed as different clinical entities, all belonging to the same clinical spectrum. Activating mutations in the *FGFR3* gene cause permanent activation of the FGFR3 regardless of the presence of its ligand, resulting in ceasing the chondrogenesis in the growth plate. The exact extent of this growth faltering will be determined by the exact nature of the activating mutation. Different activating mutations in the *FGFR3* gene may cause achondroplasia (OMIM 100800), thanatophoric dysplasia type I (OMIM 187600), which is a short-limb dwarfism syndrome usually lethal in the perinatal period, as well as hypochondroplasia (OMIM 146000) with clinical features, including short-limbed dwarfism, lumbar lordosis, short and broad bones, and caudal narrowing of interpediculate distance of lumbar spine. 

One individual with posteriorly placed maxillae, diagnosed through a lateral cephalometric radiograph, had two mutations (A213G and A223G) in exon 3 of the *FGFR3* gene in compound heterozygosity [125]. Six patients with non-syndromic isolated sagittal synostosis had a common *FGFR3* polymorphism 294C>T (Asn294Asn). The intracranial volumes were assessed with 3D CT but no statistical difference was found between them and those of healthy individuals [126]. Among eight patients with synostotic frontal plagiocephaly (unilateral coronal synostosis), three patients had *FGFR3* Pro250Arg mutation, two patients had mutations in the *FGFR2* gene, and three patients in the *Twist* gene. Two features were strongly associated with a detectable mutation in all patients with synostotic frontal plagiocephaly: asymmetrical brachycephaly (retrusion of both supraorbital rims) and orbital hypertelorism. Other abnormalities in the craniofacial region and extremities were clues to a particular mutation in the *FGFR2*, *FGFR3,* or *Twist* genes. Neither macrocephaly, degree of nasal angulation, nor the relative vertical position of the lateral canthi correlated with mutational detection [127].

### 6.6. Parathyroid Hormone-like Hormone (PTHLH) or Parathyroid Hormone-Related Protein (PTHrP)

Mutations in the *PTHLH* gene (12p11.22) cause brachydactyly type E (OMIM 613382), an autosomal dominant syndrome characterized by short stature, brachydactyly, and learning difficulties. In an experimental animal model, it was observed that higher levels of PTHrP expression coincided with the slowing of chondrocyte hypertrophy [128]. Mandibular advancement in rats increased the number of differentiated chondroblasts and triggered PTHrP expression, which retarded their further maturation to allow for more growth and subsequent increase in the cartilage volume.

## 7. Discussion

Disruptions of the biological factors regulating the molecular events taking place at the epiphyseal growth plate during the developmental time period can result in stature abnormalities [129]. Short stature is characterized as either primary or secondary when a recognizable genetic or endocrine disease is responsible for its occurrence [1,130]. Sometimes, despite the meticulously carried out diagnostic work-up no underlying cause can be unveiled, and, thus, short stature is characterized as idiopathic (ISS) [130]. It is possible that ISS constitutes a manifestation of a milder phenotypic expression of some known genetic or endocrine disease presenting with very few or even without any clinical findings and, thus, remains undiagnosed [129]. It would be of great clinical importance if there was a diagnostic tool capable of recognizing the underlying cause in such borderline cases where the physical findings are normal or close to normal.

Little is known about the correlation between the standing body height and the quantitative extent of growth of the craniofacial complex, and even less is known about the changes that are induced in the craniofacial complex in individuals with genetic conditions that cause short stature. If such a relation is established, then specific causes of short stature could be attributed to specific craniofacial abnormalities. A useful, inexpensive, widely available, and daily utilized diagnostic tool for assessing the craniofacial complex is the cephalometric radiograph, which may quantify the degree of oral and maxillofacial growth retardation. The assessment of the cephalometric radiograph requires an experienced general dentist, a specialized orthodontist or an oral and maxillofacial surgeon and this constitutes a disadvantage of the method. In addition to that, the cephalometric radiograph diagnostic method bears some inherent limitations. These limitations include image structural noise from the two-dimensional representation of a three-dimensional structure, potential errors in achieving reproducibility while obtaining successive radiographs resulting in artefacts during comparisons, errors during comparison of radiographs and potential errors during the process of cephalometric tracing, the identification of the cephalometric landmarks and reference points [131]. If each genetic cause of short stature presents with a specific pattern of cephalometric measurement aberrations, then the cephalometric radiograph could be used in guiding further genetic testing or even in setting the diagnosis on its own.

Cephalometric evaluation of children with reduced stature due to genetic causes demonstrates that these individuals exhibit abnormal growth of the craniofacial complex [132]. Craniofacial growth seems to follow the linear somatic retardation, with the individuals exhibiting reduced linear measurements and abnormal angular measurements when compared to the reference values of normal individuals. Both the maxilla and the mandible are shorter and posteriorly placed and their anteroposterior arrangement results in a skeletal class II pattern and a retrognathic profile of the face. The angular measurements in the vertical plane constitute the skeletal planes as hyperdivergent and imply a posterior/downward inclination of the mandible with a vertical growth pattern of the face, reduced posterior facial height and increased anterior facial height.

Some syndromes present with characteristic aberrations in the cephalometric measurements, whereas other genetic disorders present with similar and non-distinguishable pathologic craniofacial characteristics. For instance, in individuals with Turner syndrome, the most commonly affected craniofacial structure is the cranial base, with the posterior cranial base exhibiting reduced length and the cranial base angle being increased (increased angle of flexion), causing a flattening of the cranial base, while in individuals with achondroplasia, the most prominent manifestation is the significantly reduced posterior cranial base length and the acute cranial base angle, while milder phenotypes fall between these two conditions [24,25,35]. Thus, if studies could propose a cut-off value for the cranial base angle above which the diagnosis of Turner syndrome is probable and below which the diagnosis of achondroplasia is probable, enabling clinicians to further expand these measurements in the diagnosis of other genetic disorders solely based on their craniofacial phenotype.

Interestingly, the greater part of the existing literature on cephalometric measurements in short-statured children with genetic syndromes provides qualitative data, whereas quantitative data are required to potentially establish the above-mentioned cut-off values. Moreover, literature providing cephalometric data for individuals affected with rare genetic conditions causing short stature is scarce. Consequently, more studies providing quantitative cephalometric data in large populations of affected individuals need to be conducted.

## Figures and Tables

**Table 1:** Cephalometric data for common genetic syndromes associated with short stature. **(NA: data not available).**

GENETIC SYNDROMES ASSOCIATED WITH SHORT STATURE	DATA FROM LATERAL CEPHALOMETRIC RADIOGRAPHS			
	Skull	Anteroposterior Plane	Vertical Plane	Teeth
Achondroplasia (1 in 15,000–40,000)	Skull deformities Pneumatized frontal sinuses Significantly reduced posterior cranial base length Acute cranial base angle Significantly reduced length of the cribriform plate of the ethmoidal bone Remarkably increased anterior sphenoidal length	Posteriorly placed and smaller maxilla and an anteriorly or normally placed mandible Skeletal class III that can result in an anterior cross-bite	Reduced upper anterior facial height Posterior tilt of the nasal floor (palatal plane) High coronoid process	Maxillary incisors labially proclined
Turner syndrome (1 in 2000–3000)	Reduced posterior cranial base length Increased cranial base angle Smaller and thiner calvarium Fused cervical vertebrae Inferiorly and anteriorly placed external acoustic meatus Smaller, more delicate and less pneumatized mastoid processes Large and excessively pneumatized sphenoidal sinuses Smaller sella turcica Premature calcification of the petroclinoid ligament Reduced facial/cerebral skull ratio	Reduced length of the maxilla Reduced length of the mandible Posteriorly positioned maxilla Posteriorly positioned mandible Posteriorly positioned chin Skeletal class II Transverse plane Facial asymmetry Posterior cross-bite as a result of the transversal dimension reduction of the maxilla	Hyperdivergent skeletal planes Reduced posterior facial height Increased anterior facial height	Occlusal plane angle is remarkably tilted Maxillary incisors lingually inclined Short teeth roots Remarkably low tongue position Pharyngeal airway space is narrower in all its dimensions
Noonan syndrome (1 in 1000–2500)	NA	Class I molar relationship and class III cuspid relationship, Class I skeletal relationship Class II skeletal relationship	Vertical mandibular growth pattern with hyperdivergent planes and increased vertical angles. Both an increased and a decreased vertical overjet have been reported	Labially inclined maxillary mandibular incisors Palatally inclined maxillary incisors and labially inclined mandibular incisors Lingually inclined mandibular incisors
Idiopathic growth hormone deficiency	Reduced anterior cranial base length	Reduced maxillary length and mandibular length	Reduced anterior facial height and ramus height	NA
Prader-Willi syndrome (1 in 10,000–20,000)	Reduced cranial base angle	Skeletal class II with posteriorly placed mandible and reduced mandibular and maxillary length Skeletal class III with anteriorly placed mandible	Vertical growth direction and increased anterior facial height In cases of skeletal class III, horizontal growth direction	Soft tissue excess In cases of skeletal class III, lingually inclined mandibular incisors and labially inclined maxillary incisors
Muenke syndrome (1 in 30,000)	Decreased intracranial volume Significantly reduced anterior cranial base and skull length Increased angle between cranial base and Frankfort horizontal plane Hypertelorism Frontal bossing	Reduced length of the maxilla and midface deficiency Reduced length of the mandible Posteriorly placed maxilla Transverse plane Increased facial width Significant skeletal asymmetry	Reduced upper and lower anterior facial height Hypedivergent skeletal planes Increased gonial angle Anterior open bite	NA

**Table 2:** Cephalometric data for less common genetic syndromes associated with short stature, (NA: data not available).

GENETIC SYNDROMES ASSOCIATED WITH SHORT STATURE	DATA FROM LATERAL CEPHALOMETRIC RADIOGRAPHS			
	Skull	Anteroposterior Plane	Vertical Plane	Teeth
22q11.2 deletion syndrome	Increased cranial base angle	Posteriorly placed mandible	NA	NA
49, XXXXY syndrome (1 in 85,000–100,000)	NA	Anteriorly placed mandible	NA	Lingually inclined mandibular incisors
Catania brachydactylous type of acrofacial dysostosis	NA	No distinctive abnormalities	NA	NA
Beckwith–Wiedemann syndrome (1 in 11,000)	NA	Dental class I	Vertical growth pattern Anterior open bite	Macroglossia
Bloch–Sulzberger syndrome (1,2 in 100,000)	NA	Reduced maxillary length	Hyperdivergent skeletal planes	Lingually inclined maxillary incisors
Chronic acid sphingomyelinase deficiency (1 in 250,000)	NA	Posteriorly placed maxilla and mandible and skeletal class II	NA	Increased nasolabial angle Convex profile Retroinclination of maxillary and mandibular incisors
Cockayne syndrome (2–3 in a million)	Hypodevelopment	Posteriorly placed and shorter mandible and skeletal class II	NA	NA
Syndromic craniosynostosis with fused spheno-occipital synchondrosis (1 in 100,000)	Moderate and severe upward anterior cranial base inclination	Severe midface deficiency Higher percentage of severe Class III skeletal pattern	Severely hyperdivergent skeletal planes Severely forward condyle position	NA
Ectodermal dysplasia 1, hypohidrotic (1 in 10,000–100,000)	NA	Reduced length and posterior placement of the maxilla Anteriorly placed mandible with protruding chin Skeletal class III	Hyperdivergent skeletal planes Reduced anterior facial height Reduced upper anterior facial height	First maxillary molars located in higher positions
Ectodermal dysplasia anhidrotic or Rapp–Hodgkin syndrome	NA	Mildly to moderately reduced mandibular length with anterior mandibular placement Maxilla placed closer to the anterior cranial base	NA	NA
Ellis–Van Creveld syndrome (1 in 60,000–200,000)	NA	Skeletal class I or class II with posteriorly placed mandible Class III with anteriorly placed mandible, or posteriorly placed maxilla	Hyperdivergency of the skeletal planes, normal vertical growth direction, or even horizontal growth pattern	Mandibular and maxillary incisor retroclination Upper lip retrusion Lower lip retrusion Both concave and convex profiles have been reported
Hajdu–Cheney syndrome (less than 100 cases described)	Increased cranial base length Enlarged, elongated, and wide open sella turcica with slender clinoids	Posteriorly placed maxilla and mandible	NA	NA
Hallerman–Streiff syndrome (less than 200 people worldwide)	NA	Skeletal and dental class II due to shorter and posteriorly placed mandible	Vertical growth pattern with an opening of the gonial angle, a large anterior open bite, and an excessive increase in the lower anterior facial height	NA
Kabuki syndrome (1 in 32,000)	NA	Posteriorly placed maxilla and mandible with a skeletal Class I pattern	Increased lower anterior facial height and anterior open bite	NA
Klippel–Feil syndrome (1 in 40,000)	Fused cervical vertebrae	Skeletal class I	Vertical growth pattern	NA
Langer–Giedion syndrome (extremely rare)	NA	Posteriorly placed maxilla and mandible	NA	NA
Larsen syndrome (1 in 100,000)	Orbits positioned posteriorly relative to the anterior cranial base	Posteriorly positioned maxilla and mandible with skeletal Class III pattern Transverse plane Hypertelorism Narrow maxillary basal arch Reduced maxillary and mandibular dental arch widths	Increased vertical angles with a large Gonial angle Growth tendency of the mandible toward the posteroinferior direction	Mandibular primary incisors lingually inclined
Methylmalonic aciduria and homocystinuria (1 in 200,000)	Head rotated and bent towards the left shoulder, which is located in a lower position than the right one Horizontal planes of both maxillary bones converge towards the right	NA	NA	NA
Moebius syndrome (1 in 50,000–500,000)	NA	Posteriorly placed mandible with reduced length and skeletal class II	Increased maxillary height resulting in a vertical growth pattern	Proclined maxillary and mandibular incisors Protrusion of upper and lower lips Long upper lip
Congenital or childhood onset myotonic dystrophy type I (1 in 9000)	NA	Increased ANB angle and reduced facial angle	Hyperdivergent skeletal planes with mandibular plane angle and intermaxillary angle increased	NA
Rubinstein-Taybi syndrome (1 in 100,000 to 125,000)	Brachycephaly	Skeletal class II	NA	NA
Seckel syndrome (1 in 10,000)	Small skull with an extremely short anterior cranial base and maxillary length Differences in the morphology of the sella turcica observed between girls and boys	NA	NA	NA
Silver–Russell syndrome (1 in 30,000–100,000)	NA	Skeletal class II with posteriorly placed mandible Class I and III have also been reported	NA	NA
Simpson–Golabi–Behmel syndrome	Increased anterior cranial base length	Increased length of the maxilla and the mandible with a skeletal class III pattern	Increased lower anterior facial height	NA
Solitary Median Maxillary Central Incisor syndrome (1 in 50,000)	Hypoplastic sella turcica Cervical vertebral maturation (CVM) at stage CS2	Skeletal class III with an anterior cross-bite as a result of reduced maxillary length and anteriorly placed mandible	Vertical growth pattern	Convex profile Airway patency Maxillary and mandibular incisal proclination
Treacher–Collins syndrome (1 in 50,000)	Reduced length of both the anterior and posterior cranial base and a reduced cranial base angle	Posteriorly placed maxilla with reduced length Posteriorly placed mandible with a characteristic reduction of the mandibular length Reduced maximum ramus width	Hyperdivergent skeletal planes and increased gonial angle Both the anterior and posterior facial heights are decreased	The maxillary and functional occlusal planes are tipped upwards posteriorly
Williams syndrome (1 in 7500–18,000)	Reduced anterior cranial base length	Posteriorly placed chin	Hypedivergent skeletal planes Unusual proportion of upper to lower anterior facial height and posterior to anterior facial height	NA

## Data Availability

The original contributions presented in this study are included in the article. Please direct any additional inquiries to the corresponding author.
